# Acute kidney injury caused by venomous animals: inflammatory mechanisms

**DOI:** 10.1590/1678-9199-JVATITD-2020-0189

**Published:** 2021-08-25

**Authors:** Naila Albertina de Oliveira, Simone Cristina Cardoso, Dulce Aparecida Barbosa, Cassiane Dezoti da Fonseca

**Affiliations:** 1 Department of Nursing, Institute of Health Sciences, Paulista University (Unip), Jundiaí, SP, Brazil.; 2 Graduate Program in Nursing, School of Nursing, Federal University of São Paulo (Unifesp), São Paulo, SP, Brazil.; 3 Irmandade da Santa Casa de Misericórdia de Piracicaba, Piracicaba, SP, Brazil.; 4 Department of Clinical and Surgical Nursing, School of Nursing, Federal University of São Paulo (Unifesp), São Paulo, SP, Brazil.

**Keywords:** Envenoming, Venom, Inflammatory process, Snake, Lonomia, Scorpion

## Abstract

Either bites or stings of venomous animals comprise relevant public health problems in tropical countries. Acute kidney injury (AKI) induced by animal toxins is related to worse prognostic and outcomes. Being one the most important pathways to induce AKI following envenoming due to animal toxins, inflammation is an essential biological response that eliminates pathogenic bacteria and repairs tissue after injury. However, direct nephrotoxicity (i.e. apoptotic and necrotic mechanisms of toxins), pigmenturia (i.e. rhabdomyolysis and hemolysis), anaphylactic reactions, and coagulopathies could contribute to the renal injury. All these mechanisms are closely integrated, but inflammation is a distinct process. Hence, it is important to improve our understanding on inflammation mechanisms of these syndromes to provide a promising outlook to reduce morbidity and mortality. This literature review highlights the main scientific evidence of acute kidney injury induced by bites or stings from venomous animals and their inflammatory mechanisms. It included observational, cross-sectional, case-control and cohort human studies available up to December 2019. Descriptors were used according to Medical Subject Headings (MeSH), namely: “Acute kidney injury” or “Venom” and “Inflammation” on Medline/Pubmed and Google Scholar; “Kidney disease” or “Acute kidney injury” on Lilacs and SciELO. The present review evidenced that, among the described forms of renal inflammation, it can occur either directly or indirectly on renal cells by means of intravascular, systemic and endothelial hemolysis, activation of inflammatory pathway, as well as direct action of venom cytotoxic components on kidney structures.

## Background

Either bites or stings of venomous animals are considered a public health problem in tropical countries. Acute kidney injury (AKI) is the main complication resulting from accidents caused by animal toxins and it is related to higher mortality rates [1, 2]. In the Brazilian Amazon, mortality rate from snakebites has been estimated at 0.51%, which is 10 times higher than the estimated global average [2]. *Bothrops*, *Crotalus*, *Lachesis* and *Micrurus* are the most important genus of snakes from the medical point of view in Brazil, with genus *Bothrops* (pit vipers) being responsible for most of the bites (90%) and deaths related to snakes (0.3%) in the Brazilian Amazon [2]. AKI incidence can reach up to 50% in victims of bites or stings of venomous animals [1, 2].

According to the organization Kidney Disease: Improving Global Outcomes (KDIGO), AKI is defined by serum creatinine increase ≥ 0.3 mg/dL (26.5 µmol/L) within 48 hours or increase ≥ 1.5 times baseline or urine volume < 0.5 mL/kg/h for 6 hours [3]. The early identification of this syndrome using KDIGO tool contributes to rapid intervention with the aim at reducing morbidity and mortality.

Bee melittin, larvae and scorpion venoms are main animal toxins involved in AKI induced by stings whereas snake venoms are main toxins inoculated by bites [4]. It is also necessary to differentiate between inflammation and other pathways of AKI mechanisms due to venomous toxins. Inflammation is one the most important mechanism that affects hemodynamic kidney changes.

Hemolysis and rhabdomyolysis have been described as precursors of this AKI pathophysiology. Upon contact with the bloodstream, the toxin activates immune and inflammatory mechanisms that exacerbate the expression of cytokines and stimulate coagulation factors resulting in hemoglobin degradation and pigmenturia formation [5]. These compounds come into contact with renal parenchyma and cause intratubular obstruction and direct toxicity, corroborating the decrease in glomerular filtration rate and renal failure [6].

Therefore, this review aims at highlighting the main evidence on inflammatory and oxidative pathophysiological mechanisms of AKI induced by venomous animal bites or stings while contributing to the early identification of AKI by the multidisciplinary health team.

## Methods

This work carried out an integrative literature review from December 2019 to September 2020, comprising the following stages: (*i*) elaboration of theme and guiding question, (*ii*) data selection for inclusion-exclusion criteria analysis, (*iii*) search in databases, and (*iv*) critical analysis of material to be used in study construction and results interpretation. As means to guide the search for scientific studies while aiming at best scientific evidence for integrative review, PICO (patient/problem, intervention, comparison/control, and outcomes) strategy was used [7].

The search was made into four steps: (*i*) problem identification; (*ii*) formulation of relevant and specific issue; (*iii*) search for scientific evidence; and (*iv*) evaluation of available evidence. For this study, PICO structure to define the guiding question was: P = venom from venomous animals; I = injury to renal parenchyma; C = inflammatory mechanisms; O = acute kidney injury. Based on this tool, the research question was: ‘what is the impact of either bites or stings from venomous animals on renal parenchyma and their relationship with inflammatory mechanisms in acute kidney injury?’

Following this question, publications were searched and selected in line with recommendations from Preferred Reporting Items for Systematic Reviews and Meta-Analyses (PRISMA) [8]. Limited to English and Portuguese while including articles published until September 2020, the search considered the following databases: Medline/Pubmed, SciELO and Lilacs.

The following descriptors were used according to Medical Subject Headings (MeSH): “Acute kidney injury” or “Venom” and “Inflammation” on both Medline/Pubmed and Google Scholar, “Kidney disease” or “Acute kidney injury” on both Lilacs and SciELO. Searched in associated way, those descriptors were united via Boolean operator ‘AND’ consistent with the following equation: {(acute kidney injury) [mesh] AND (venom) [mesh]} AND (inflammation).

**Figure 1.**
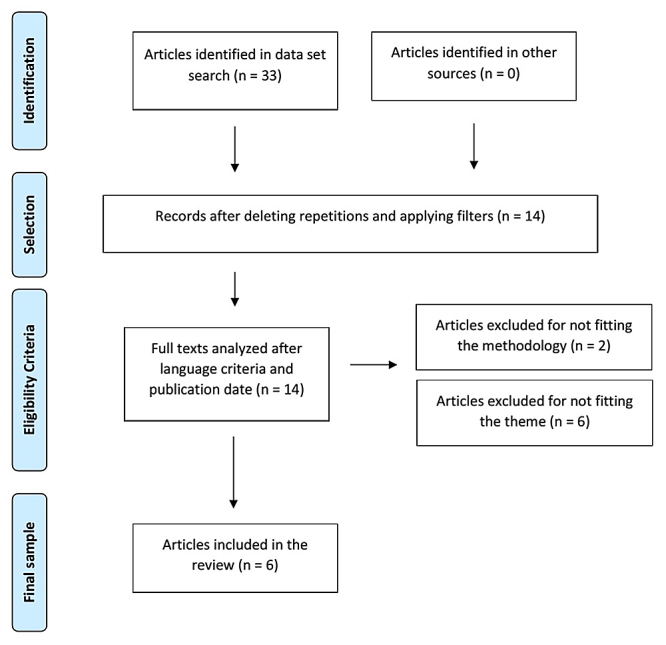
Description of selected studies (São Paulo, Brazil, 2021).

Inclusion criteria comprised: free online full-text articles with original experiments, pre-clinical studies published from 2010 to 2020, studies of all characters significantly approaching the investigated subject in terms of acute kidney injury (AKI) in incidents with venomous animals. Exclusion criteria were: deficient information articles, integrative review articles addressing different health areas about venomous animals, and correlation with AKI following the research criteria as sketched in Figure 1.

Articles found were classified according to research type and evidence level, namely:

- Level 1: evidence from systematic review or meta-analysis;
- Level 2: evidence from at least one well-designed randomized controlled clinical trial;
- Level 3: evidence from well-designed clinical trials without randomization;
- Level 4: evidence from well-designed cohort and case control studies;
- Level 5: evidence from systematic review of descriptive and qualitative studies;
- Level 6: evidence from a single descriptive or qualitative study;
- Level 7: evidence from authorities’ opinion and/or expert committees’ report.

Initially selected by three reviewers, articles were subsequently verified by a fourth reviewer in order to guarantee process specificity and quality. All searched studies were compiled in Rayyan systematic review processing software (www.rayyan.qcri.org) [9] for better efficiency and impartiality during the selection process when reading them in full.

Through inclusion criteria filtering and careful analysis, 33 articles were found. After applying inclusion/exclusion and duplication criteria, 19 articles were excluded and the 14 articles left were read in full. After this reading, eight articles not meeting eligibility criteria were excluded thus remaining six articles to comprise the final sample.

For data presentation and analysis, the following aspects were taken into account: ‘authorship’, ‘year’, ‘title’, ‘periodic’ and ‘focus’. Variables presentation was organized and simplified according to the flowchart in Figure 1.

In searched databases 33 articles were found, which were selected and whose abstracts were read in full. Accordingly, 14 articles were selected as their content included the research aim. From reading their abstracts, eight articles were excluded because: (*i*) the article did not address any aspect about venom from bees, scorpions, *Lonomia* or snakes (n = 1), (*ii*) the article was a case report on an accident with venom without definite specification about the venom (n = 1), (*iii*) the articles were reviews about accident reports with bee stings in children (n = 2), and (*iv*) the articles dealt with positive results in using bee venom as medication (n = 4). After analytically reading articles in full followed by exclusions, the sample consisted of six articles meeting all inclusion criteria as listed in Table 1 and detailed in Table 2.

**Table 1.** Article distribution according to title, authors, country, year and journal (São Paulo, Brazil, 2020).

(N)	Title	Authors	Year	Country	Journal
(1)	Myotoxicity and nephrotoxicity by *Micrurus* venoms in experimental envenomation [33]	Adolfo Rafael de Roodt, Néstor Rubén Lago, Roberto Pablo Stock	2011	USA	Toxicon
(2)	Hematological alterations and splenic T lymphocyte polarization at the crest of snake venom induced acute kidney injury in adult male mice [35]	Farhat Nasim, Sreyasi Das, Roshnara Mishra, Raghwendra Mishra	2017	USA	Toxicon
(3)	Determination of the sub-lethal nephrotoxic dose of Russell's viper (*Daboia russelii*) venom in Wistar rats [47]	Eranga S. Wijewickramaa, Ishani Kurukulasooriya, Mangala Gunatilake, Amarathunga AH. Priyanic, Ariaranee Gnanathasana, Indika Gawarammanad, Geoffrey K. Isbistere	2018	USA	Toxicon
(4)	Induction of two independent immunological cell death signaling following hemoglobinuria-induced acute kidney injury: in vivo study [14]	Rana Dizajia, Ali Sharafib, Jalal Pourahmada, Mohammad-Amin Abdollahifard, Hossein Vatanpoura, Mir-Jamal Hosseinib	2019	USA	Toxicon
(5)	Acute *Lonomia obliqua* caterpillar envenomation-induced physiopathological alterations in rats: evidence of new toxic venom activities and the efficacy of serum therapy to counteract systemic tissue damage [15]	Markus Berger, Walter Orlando Beys-da-Silva, Lucélia Santi, Iuri Marques de Oliveira, Patrícia Mendes Jorge, João Antônio Pêgas Henriques, David Driemeier, Maria Aparecida Ribeiro Vieira, Jorge Almeida Guimarães	2013	USA	Toxicon
(6)	Mechanisms of acute kidney injury induced by experimental *Lonomia obliqua* envenomation [16]	Markus Berger, Lucélia Santi, Walter O. Beys‐da‐Silva, Fabrício Marcus Silva Oliveira, Marcelo Vidigal Caliari, John R. Yates III, Maria Aparecida Ribeiro Vieira, Jorge Almeida Guimarães	2014	USA	Arch Toxicol

**Table 2.** Details of selected articles for integrative review: research outline, objective, main results and conclusion (São Paulo, Brazil, 2020).

Research outline	Objective	Main results	Conclusions
(1) Experiments were performed in laboratory animals with Wistar rats (250 g) and CF-1 mice that received venoms from *Micrurus fulvius* and *M. nigrocinctus*. Parameters evaluated: lethality, coagulation changes, hemorrhagic process assessment, phospholipase activity, hemolysis, uremia, inflammation and edema, as well as histopathological analysis.	To evaluate *in vivo* skeletal muscle changes and their association with renal lesions in rats intramuscularly injected with venom from different species of *Micrurus* from South, Central and North America.	Kidneys of rats injected with either *M. fulvius* or *M. nigrocinctus* venoms exhibited nuclear fragmentation, edge destruction, basement membrane rupture and epithelial exfoliation of tubular cells, presence of granular changes and tubules thickening. Presence of abundant protein material and granular cylinders was evidenced in tubules of different nephron sections. Both glomerular congestion and intracapillary thrombi presence were observed in glomeruli of mice injected with these two venoms.	Although renal lesions have not been described in clinical cases of *Micrurus* envenoming, the potential for nephrotoxicity of these venoms should be considered since the kidneys of animals exposed to *M. fulvius* or *M. nigrocinctus* venoms presented lesions consistent with extensive tubular necrosis, brush border destruction, basement membrane rupture and epithelial exfoliation of tubular cells, granular plaster and tubules thickening.
(2) In this study, Swiss mice were the experimental model. Hematological changes, polarization of associated splenic T cells were analyzed in order to investigate the immune response to Russell viper venom (RVV) using acute kidney injury (AKI) induction model.	To investigate the immune response to Russell viper venom in acute kidney injury induction model in a murine experimental model.	In the group with acute kidney injury caused by snake venom, findings such as oliguria, urinary microprotein with significant elevation, decreased urinary creatinine and creatinine clearance were confirmed in comparison with the control group. Hematological analyzes revealed significant neutrophilic leukocytosis, favoring a state of acute inflammation in the group of acute kidney injury induced by snake venom (SAKI). The splenocyte immunophenotyping study showed a significant decrease in CD4 + / CD8 + ratio with a significant increase in the regulatory helper (CD25 + FoxP3 +) and cytotoxic subset of T cells. In addition, regulatory T cells in CD25-FoxP3 + reservoir were also found to be significantly elevated in the SAKI group compared to the control.	Results from the present study clearly indicated a state of acute inflammation and polarization of splenic T cells towards the regulatory subset at the crest of SAKI. The findings of this research also support the concept of circulatory involvement and splenic inflammatory and immunological mediators in pathogenesis and/or repair phase of acute kidney injury induced by snake venom, which was otherwise attributed to the direct toxic effect of the venom.
(3) In this study, Wistar rats received increasing doses of intraperitoneal Russell viper venom to investigate acute kidney injury (AKI) by measuring creatinine and examining renal histology.	To investigate acute kidney injury (AKI) by measuring creatinine (1.5-fold increase in serum creatinine above baseline) and examining renal histology after administration of increasing doses of Russell viper venom.	Increase in serum creatinine occurred only with 0.4 mg/kg venom dose; acute tubular necrosis, glomerular necrosis, cortical necrosis and interstitial inflammation were observed with venom doses ≥ 0.25 mg/kg.	Despite widely used as standard biomarker for AKI diagnosis, serum creatinine is considered a delay and relatively sensitive biomarker for detecting kidney injury. The study confirmed an increase in serum creatinine significant enough to diagnose AKI that showed histological evidence of nephrotoxicity. In contrast, 5 out of 7 rats that demonstrated a significant increase in serum creatinine showed histological evidence of nephrotoxicity.
(4) *In vivo* study used male Balb/c mice subcutaneously inoculated with *H. lepturus* venom. After 1 and 7 days, urinalysis, stereological evaluations and gene expression of Ngal, Tnf-α, Tlr-4, Ripk3, Mlkl and Acsl4 were performed. Analyzes were performed through real-time PCR.	To evaluate the potential role of necroptosis and ferroptosis in AKI induced by *H. lepturus*.	The study pointed that overloading in Ngal renal expression (nephrotoxicity biomarker) refers to overexpression of Tnf-α, Tlr-4, Ripk3 and Mlkl genes in kidneys treated with venom; it was also observed that malondialdehyde (MDA) level was increased in a dose-dependent manner similar to Acsl4 gene expression, thus suggesting a major role of ferroptosis in hemoglobinuria-mediated ARI after envenoming while increased transcription of Tlr-4 and Tnf-α receptor may cause phosphorylation of Ripk3-Mlkl complex, collapse of membrane potential and DAMPs release that intensified inflammation cytokines in the kidney.	The study assumes that coexistence of two separate pathways of regulated necrosis and inflammatory environment provide a promising perspective in the prevention and treatment of hemoglobinuria-induced ARI after envenoming. Findings of this research revealed that AKI induced by hemoglobinuria is associated with the expression of inflammatory cytokines, renal cell death and overexpression of Ngal gene. This study emphasizes the role of immunogenic cell death attributable to regulated necrosis during venom-induced ARI. In this regard, prevention of regulated necrosis and inflammation seem beneficial for AKI treatment.
(5) Wistar rats received *L. obliqua* venom; after the experiment, acute effects were observed after the administration of injected subcutaneous venom; biochemical, hematological, histopathological, myotoxic and genotoxic alterations were described.	To understand systemic pathophysiological mechanisms of *L. obliqua* envenoming.	Hematological findings were consistent with hemolytic anemia and neutrophilic leukocytosis. Histopathological changes were mainly related to hemorrhage and inflammation in subcutaneous tissue, lung, heart and kidneys. Myoglobin cylinders were also detected in renal tubules. Increased levels of creatine kinase and creatine kinase-MB were correlated with myocardial necrosis as observed in vivo and confirmed myotoxicity detected in vitro in isolated long extensor muscles of fingers. Significant DNA damage was observed in kidneys, heart, lung, liver and lymphocytes.	Data reveal important biochemical, hematological and histopathological changes, suggesting damage occurrence to multiple organs, hemorrhagic disorders, AKI. The study demonstrated myotoxic, cardiotoxic and genotoxic activities.
(6) In this study, *L. obliqua* venom was subcutaneously injected into Wistar rats and renal function was examined, besides morphological and biochemical parameters.	To evaluate possible mechanisms involved in renal dysfunction pathogenesis due to *L. obliqua* envenoming.	*L. obliqua* envenoming causes acute tubular necrosis, which is associated with renal inflammation; formation of hematic molds, resulting from intravascular hemolysis; increased vascular permeability and fibrosis. Envenomed kidneys increase the expression of proteins involved in cell stress, inflammation, tissue damage, heme-induced oxidative stress, coagulation and activation of the complement system.	Mechanisms of *L. obliqua*-induced AKI are complex and involve mainly glomerular and tubular functional impairment and vascular changes.

### Lonomia

Caterpillars from *Lonomia* genus have their bodies covered with bristles that inject venom when touched by victims [10, 11]. As their natural habitat is deep forest trees, deforestation has forced these animals to migrate closer to human dwellings, thus increasing the number of accidents [11-14].

Clinical manifestations of *Lonomia obliqua* envenoming include hemorrhage and acute kidney injury (AKI) whereas clinical conditions from those accidents are characterized by contact site burning, headache, fever, vomiting, and hemorrhagic diathesis, which can be fatal [10-12]. In cases of envenoming from *Lonomia obliqua*, victims may also present systemic hemorrhage secondary to disseminated intravascular coagulation [15-17].

AKI associated with *Lonomia* venom has been mainly reported in Brazil [13, 18, 19]. Early administration of antivenom in rats prevented hemorrhagic manifestations induced by *Lonomia* venom, which causes serum creatinine increase [13, 20].

AKI mechanisms due to *Lonomia obliqua* venom are direct nephrotoxicity, intravascular, systemic and endothelial hemolysis, activation of inflammatory pathway, hypotension, increased expression in renal tissue of proteins involved in cellular stress, inflammation, heme-induced oxidative stress, coagulation and activation of complement system [15-17]. Renal histology showed glomerular fibrin deposition as well as ischemia and tubular atrophy [15].

Studies observed in this review showed that animals receiving *Lonomia obliqua* venom produce dark brown urine, indicating hematuria and/or hemoglobinuria occurrence. Presence of erythrocytes, epithelial cells and leukocytes was also observed in urine sediment and studies also showed uremia and hyperuricemia, thus suggesting renal failure [15-17].

In AKI, venom can induce a sudden loss of basic renal functions such as filtering and excreting abilities, urinary concentration and changes in body fluid homeostasis. Main cytotoxic effects from venom toxins cause renal hypoperfusion which is, therefore, an important underlying mechanism as it shows glomerular fibrin deposition and hemodynamic instability (systemic hypotension and increased renal vascular permeability) due to kallikrein-kinin system activation during envenoming [15, 17].

Entailing proteolytic enzymes and their respective substrates, the kallikrein-quinine system can generate potent vasoactive and pro-inflammatory molecules that are involved in controlling blood pressure, vascular permeability, contraction or relaxation of vascular smooth muscle cells and pain. Both tissue and plasma kallikreins are essential elements in this system as they generate kinins through proteolytic cleavage of kininogens [15, 17].

At the same time, kallikrein can also directly convert FXII into its active form FXIIa, leading to kinin system self-activation and thrombi formation by intrinsic route [21-25]. Once released, quinines exert most of their biological effects by activating two types of quinine receptors, namely: bradykinin B1 receptor (B1R) and bradykinin B2 receptor (B2R). Constitutively expressed in majority of tissues, B2R has greater affinity with peptides bradykinin (BK) and Lys-BK [17, 25]. In contrast, B1R exhibits high affinities for quinine metabolites des-Arg9-BK and Lys-des-Arg9-BK. B1R is not expressed under normal conditions, being induced after inflammatory, infectious or traumatic stimuli [17, 25].

Considerably, kallikrein and kinin receptors (B1R and B2R) are involved in several processes relating inflammation such as atherosclerosis, airway inflammation, diabetic neuropathy, inflammatory bowel disease, neuropathic pain, and cerebral infarction (stroke) [26, 27]. *In vivo* studies showed that blockade of distinct members of kallikrein-quinine system reduced vascular leakage, inflammation and thrombus formation in different experimental models [28].

Glomerular filtration rate and electrolyte imbalance are reduced as observed in *Lonomia* envenoming. Berger et al. [17] demonstrated that *L. obliqua* venom has high quininogenase activity, able to release low molecular weight kininogen bradykinin, leading to significant decrease in blood pressure *in vivo*. They showed the venom can directly activate pre-kallikrein plasma, acting as a probable kallikrein builder, thus initiating kallikrein-kinin system formation. Bradykinins (especially B1R) release is then activated, initiating inflammatory cascade induced by vasorelaxant effect through B2R receptor, which is converted into renal vasoconstrictor response by bradykinin metabolite des-Arg9-BK by B1R receptor.

These authors [17] also showed that *L. obliqua* envenoming triggers several hemostatic disorders such as consumption coagulopathy signs and incoagulable blood, increased clotting time, reduced levels of fibrinogen, and platelet hypoaggregation. Besides its pro-coagulant effect in plasma, *L. obliqua* venom can also induce pro-coagulant and pro-inflammatory molecules expression as tissue factor (FT), IL-6 and IL-1β in endothelial and smooth muscle vascular cells, contributing to activate coagulation cascade and subsequent incoagulability of smooth muscle vascular cells [23, 29].

In fact, in the experimental model used in [17], envenomed animals showed activity increase in renal filtration rate while smooth muscle vascular cells stimulated by *Lonomia obliqua* venom *in vitro* exhibited increase in kallikrein generating activity, changing cells to pro-coagulant profile. This study suggests the use of aprotinin as a pharmacological agent neutralizing kallikrein and consequent blocker for bradykinin release and inflammatory cascade.

It is worth mentioning that *in vitro*, when incubated with human fibroblasts or endothelial cells in culture, *L. obliqua* venom induces several pro-inflammatory cytokines production, including TNF-α and IL-1β, while it also activates nuclear factor-κB (NF-κB) and increases inflammatory enzymes expression such as cyclooxygenase-2 (COX-2), inducible nitric oxide synthase (iNOS), hemeoxygenase (HO-1) and matrix metalloproteinases (MMPs) [30]. With respect to tissue oxidative stress *in vivo*, kidneys of animals envenomed by *Lonomia obliqua* showed increased levels of peroxide, NO, MMPs and reduced levels of reduced glutathione (GSH).

These changes were also accompanied by tubular lesions, accumulation of pro-inflammatory cytokines and release of cytokines such as TNF-α, which is a known expression inducer of kininogen and bradykinin B1R receptor [23]. Collectively, all evidence points kininogen-kallikrein-BK-B1R / B2R axis activation during *L. obliqua* envenoming as blocking the initial stage of this pathway with aprotinin improves renal and vascular function.

### Snakes

Due to its high morbidity and mortality, snake envenoming is a significant public health problem in tropical countries [31-33], especially in rural areas. Snakebite accidents are generally related to weather and countryside work [4, 33]. With recognized death risk, their bites are emergencies typically leading to the following effects: local tissue damage, bleeding, coagulopathies, and shock. However, there is clinical and experimental evidence of venoms from different snakes that cause acute kidney damage [4, 33-36]. While envenoming refers to components causing neurotoxic manifestations, other other systemic manifestations are equally present [37], whose pathways are reviewed in [38].

With most studies using snake venoms, phospholipase A2, metalloprotease and sphingomyelinase are among enzymes in animal toxins contributing to renal toxicity. Phospholipase A2 (PLA2) catalyzes hydrolysis of phosphoglycerides at sn-2 acyl bond and it is divided into Class I (found in snakes from Elapidae family), Class II (in venoms of snakes in Viperidae and Crotalidae families), and Class III (found in venoms of bees *A. mellifera* and Gila monsters *Heloderma suspectum*) [39]. Playing key role in cellular injury by mediating inflammatory response, PLA2 toxic effects on biological membranes occurs via charge and van de Waals interactions leading to membrane destabilization. By augmenting membrane lipid hydrolysis, PLA2 increases membrane permeability and leads to cell lysis. While cellular exposure to PLA2 decreases membrane integrity, it increases susceptibity to H_2_O_2_ toxicity [40].

Being zinc dependent endopeptidases, metalloproteases are in venoms of snakes in Viperinae and Crotalinae subfamilies. By degrading extracellular matrix proteins while disrupting cellular matrix and cellular adhesion, aforesaid enzyme cleaves cell surface receptors and activates chemokines as well as cytokines [41]. Metalloprotease can induce apoptosis of vascular endothelial cells [42] whereas zinc metalloproteases cut glutathione-s-transferase tumor necrosis fusion protein to liberate active tumor necrosis factor [43]. Metalloproteases activate chemokine and cytokine to generate effects on leukocyte migration and inflammation [44]. Vital to both tissue remodeling and repair, metalloproteases (along with angiotensin II blocker) regress glomerular sclerotic lesion in glomerulo-sclerosis [45].

*Bothrops* are the most common snakes found in Latin America [4]. Its venom is predominantly hemotoxic and proteolytic, which means that although hemorrhage is generally the main cause of death, acute kidney damage related to the bite of this snake species is an important, potentially fatal clinical complication, as well as leading to chronic kidney disease [4].

Nephrotoxicity from snake venom can have several origins such as impaired perfusion due to intravascular coagulation, direct action of venom cytotoxic components on renal structures, and either hemoglobin or myoglobin deposits onto proximal and distal renal tubule. Another possible renal failure cause refers to cardiotoxins and nephrotoxic components in these animals venom [35, 37]. Some studies evaluated dissimilar venom fractions in different animals and their findings point to changes in renal hemodynamics as well as proximal and distal renal tubular degeneration [33, 35]. In animal studies, kinetic evaluation of urine has also proven changes in volume as well as in creatinine, microprotein and other markers of kidney damages from this venom [33, 35-37].

As American members of Elapidae family, coral snakes comprise a taxonomic group of more than 120 species and subspecies represented by genera *Leptomicrurus*, *Micruroides* and *Micrurus*, with the latter accounting for envenoming over the entire American continent [33, 46]. *Micrurus* venom causes muscle strength loss and peripheral-originated respiratory paralysis. Neurotoxic signals due to these venoms result from postsynaptic action at terminal plaque receptors level (α-neurotoxins, e.g. *Micrurus frontalis*) and/or acetylcholine release inhibition due to motor nerve endings (presynaptic PLA2, β-neurotoxins, e.g. *Micrurus corallinus*) and/or muscle fiber membranes depolarization (myotoxic A2 phospholipases, possibly other cytotoxins, e.g. *Micrurus nigrocinctus* or *fulvius*) [33, 46].

When intramuscularly injected, all venoms increased uremia levels compared to controls 24 h after injection, with the greatest increase observed with *M. pyrrhocryptus* and *M. fulvius* venoms. Elevated uremia is a robust renal failure indicator [46, 47] since plasma urea is elevated above baseline when glomerular filtration is below 50%. Renal dysfunction basis was established through histological analysis, which revealed that *M. fulvius*, *M. nigrocinctus* and (to lesser extent) *M. pyrrhocryptus* could cause major renal damage. Rat kidneys treated with these venoms had extensive tubular necrosis of epithelial cells, with basement membrane rupture and lumen epithelial exfoliation. Tubular epithelia thickened while protein material and granular plaster presence (including cylinders in tubules) was most notable in rats injected with *M. fulvius* and *M. nigrocinctus* venoms.

Models were observed in proximal and distal tubules, Henle loops, and collecting ducts. In some tubules, molds completely blocked the lumen. Glomeruli showed congestion and intracapillary thrombi presence. Rat kidneys injected with other venoms were only slightly affected, even at venom doses close to lethal values [46, 47].

Nephrotoxicity from snake venom can have several origins such as impaired perfusion due to intravascular coagulation [48, 49], direct action of cytotoxic venom components on renal structures [50, 51], and hemoglobin or myoglobin deposits in renal tubules [52, 53]. Concerning *Micrurus* venoms studied, intravascular coagulation does not seem to be involved in renal damage as these venoms did not exhibit coagulant activity in plasma while no evidence of disseminated intravascular coagulation was found in histological studies. However, it is worth mentioning that another possible cause of renal failure refers to myotoxic and nephrotoxic components of some cardiotoxins from elapid venoms [53].

In their study, Tchaou et al. [54] emphasize that viper venoms are liable for 15% envenomings and bring hemorrhagic and necrotizing complications involving several organs as well as direct toxic action of the venom on the renal parenchyma [54]. In Russel viper envenoming, acute kidney damage and nephrotoxic effects have been related to Russel’s viper venom (RVV7), which can cause hemodynamic changes and primary proximal and distal renal tubular degeneration [36, 54, 55]. In a cell culture study with proximal tubular cells, RVV-7 led to reduced cell viability, necrosis and increased LDH while basic protein isolated from RVV-7 might induce renal tubular necrosis in mice [56].

Likewise, thrombotic microangiopathy is expected to play a role in kidney damage from Russell’s viper bites. Despite the absence of arterial thrombosis, there were microthrombi in glomerular capillaries in some kidney samples from animals exposed to RVV7, thus suggesting thrombotic microangiopathy influence as evidence corroborating inflammatory and nephrotoxic process in AKI [55].

Regardless of the species, snake venom contains several proteolytic toxins causing systemic and renal hemodynamic changes, which also have thrombin-like action and fibrinolytic activity. These toxins are responsible for degrading all types of matrix proteins, activating chemokines and cytokines, as well as inducing apoptosis of vascular cell adhesion, activating clotting factors, inhibiting platelet aggregation, and inducing local symptoms at bite site [4].

### Scorpion

Scorpionism is the envenoming from the presence of toxins in scorpion venom as inoculated in the victim through the inoculating device (stinger or telson). With respect to human accident with venomous animals, scorpionism has the second morbidity incidence worldwide [57]. Scorpionic accidents are relevant due to their high occurrence frequency and severity, especially in children, the elderly and immunosuppressed victims [34]. The related nephrotoxicity is characterized as one of the most critical and lethal effects from animal venoms [58, 59]. Nephrotoxic actions have been reported for envenoming by scorpions of, for instance, species *Hemiscorpius lepturus* [60] and *Androctonus australis* [5].

Toxic venom components can directly or indirectly act on renal cells, causing mesangiolysis, glomerulonephritis, vasculitis, interstitial nephritis and nephrosis, cortical necrosis and tubular necrosis, in addition to vascular hypoxia and renal infarction. Aforesaid changes contribute to developing renal complications due to envenoming [1].

Tubular necrosis is pointed [61] as the main pathological change due to scorpion envenoming. This type of lesion reinforces urinary flow increase and intensifies diuresis while contributing to reduce electrolytic reabsorption, with increased sodium, potassium and chloride excretion.

Venom inoculation has been demonstrated [22, 55] to yield complex effects on sodium channels, especially altering electrolytes transport in renal tubules, leading to catecholamines and acetylcholine release (mediators causing serious manifestations due to parasympathetic or sympathetic effects) while intensifying tubular injury due to deleterious consequences from higher cytosolic Ca^2+^ concentration. In scorpionic accidents, pro-inflammatory cytokines and mediators - including interleukins (ILs), tumor necrosis factor (TNFα), nitric oxide (NO), platelet aggregation factor (PAF), catecholamines, prostaglandins, kinins, leukotrienes, angiotensin (AII) and endothelin (ET) - become elevated [58, 59].

At clinical level, AKI with renal failure has been observed in scorpion toxin envenoming, without hypotension and associated insults such as rhabdomyolysis, hemolysis or disseminated intravascular coagulation, with the finding that it would suggest direct nephrotoxicit [61]. Linked to hemolysis, hemoglobinuria and proteinuria, this toxin promotes important changes in the acute phase of kidney injury, which is characterized by damage to vascular structures, glomerular and tubular cells [61].

By reasoning that AKI is induced by excessive inflammation and cell death, Dijazi et al. [15] pointed that regulated cell death (necroptosis and ferroptosis) is involved in hemoglobinuria-induced AKI. Exact mechanisms of AKI induction are still unknown; however, according to Dijazi et al. [15], necroptosis induces inflammatory cytokines release such as Tnf-α and Tlr-4. Inflammatory environment, in turn, will largely increase Ripk3 and Mlkl genes in kidney due to hemoglobinuria; thus, necroptosis induction in cells will occur due to high concentration of venom, heme and expression of Tnf-α, Tlr-4, Ripk3 and Mlkl. In the experimental model used in [55], envenomed animals showed significant Tnf-α, Tlr-4, Ripk3 increases in groups exposed to 5 and 10 mg-venom/kg-body treatments. However, it was demonstrated that positive regulation is dose dependent, significantly increasing the damage according to the administered amount. This mice exposure to *Hemiscorpius lepturus* venom revealed that the venom can be found in renal tissue associated with morphological damage and renal dysfunction [15].

Dijazi et al. [15] further suppose that the natural toxin induced AKI through vasoactive effects mediated by disturbance in PLA-2 enzyme function and immune response involved in hemolysis and cytotoxicit. In turn, PLA-2 enzyme plays a fundamental role in inflammation, activating arachidonic acid that leads to eicosanoids (i.e. prostaglandins and leukotrienes) generation. PLA-2 also stimulates hypothalamic-pituitary-adrenal axis in order to produce adrenocorticotropic hormone, corticosteroids, vasopressin and acute phase proteins, while causing local manifestations at the sting site as well as hemodynamic changes [62].

In scorpion sting, aforesaid mechanism results from large amounts of hemoproteins released in extracellular space where hemoglobin oxidation occurs. In turn, after hemolysis there might be high concentration of ferric Hb (Fe^3+^), free heme group release and, thus, acceleratting the production of free radicals that accumulate in renal cortex, promoting toxic action [55].

High iron instability in heme structure is the critical factor in ROS production, which is related to Fenton reaction, NADPH oxidase and mitochondria sources [63], thus causing necrosis through TNF-α, TLR-4 and MyD88 activation. In turn, MLKL protein phosphorylation by RIPK3 receptor provides ion channels permeability while inducing membrane rupture, thus increasing ROS production and strongly inducing the immune system [16].

Alves et al. [64] demonstrated that *T. serrulatus* venom also increased PP and RVR probably due to direct vasoactive action, as evidenced by experiments on mesenteric vascular bed, whose results suggest venom effect on α1-adrenergic receptors. These receptors are abundantly found in kidneys, in afferent and efferent arterioles [64].

## Conclusion

Animal venoms have a high potential to induce AKI when inoculated in humans. Toxins from snakes, scorpions and *Lonomia* lead to significant changes in terms of perfusion pressure (PP), renal vascular resistance (RVR), urinary flow (UF), glomerular filtration rhythm (GFR), and sodium, potassium and chloride electrolyte excretion. Among described forms of renal inflammation, action can directly or indirectly occur on renal cells via intravascular, systemic and endothelial hemolysis, inflammatory pathway activation, as well as direct action of venom cytotoxic components onto kidney structures. Progressive kidney damages can ultimately lead to mesangiolysis, glomerulonephritis, vasculitis, interstitial nephritis and nephrosis, cortical necrosis and tubular necrosis, besides vascular hypoxia and renal infarction. Scientific knowledge on this issue must be expanded as only few studies in humans have reported AKI incidence and inflammatory mechanisms after exposure to these venoms.
